# Systemic and Intra-Habenular Activation of the Orphan G Protein-Coupled Receptor GPR139 Decreases Compulsive-Like Alcohol Drinking and Hyperalgesia in Alcohol-Dependent Rats

**DOI:** 10.1523/ENEURO.0153-18.2018

**Published:** 2018-07-02

**Authors:** Jenni Kononoff, Marsida Kallupi, Adam Kimbrough, Dana Conlisk, Giordano de Guglielmo, Olivier George

**Affiliations:** 1 Department of Neuroscience, The Scripps Research Institute, La Jolla, CA 92037

**Keywords:** alcohol use disorder, compulsivity, habenula, orphan receptor, pain, withdrawal

## Abstract

GPR139 is an orphan G protein-coupled receptor (GPCR) that is expressed mainly in the brain, with the highest expression in the medial habenula. The modulation of GPR139 receptor function has been hypothesized to be beneficial in the treatment of some mental disorders, but behavioral studies have not yet provided causal evidence of the role of GPR139 in brain dysfunction. Because of the high expression of GPR139 in the habenula, a critical brain region in addiction, we hypothesized that GPR139 may play role in alcohol dependence. Thus, we tested the effect of GPR139 receptor activation using the selective, brain-penetrant receptor agonist JNJ-63533054 on addiction-like behaviors in alcohol-dependent male rats. Systemic administration of JNJ-63533054 (30 mg/kg but not 10 mg/kg, p.o.) reversed the escalation of alcohol self-administration in alcohol-dependent rats, without affecting water or saccharin intake in dependent rats or alcohol intake in nondependent rats. Moreover, systemic JNJ-63533054 administration decreased withdrawal-induced hyperalgesia, without affecting somatic signs of alcohol withdrawal. Further analysis demonstrated that JNJ-63533054 was effective only in a subgroup of dependent rats that exhibited compulsive-like alcohol drinking. Finally, site-specific microinjection of JNJ-63533054 in the habenula but not interpeduncular nucleus (IPN) reduced both alcohol self-administration and withdrawal-induced hyperalgesia in dependent rats. These results provide robust preclinical evidence that GPR139 receptor activation reverses key addiction-like behaviors in dependent animals, suggest that GPR139 may be a novel target for the treatment of alcohol use disorder, and demonstrate that GPR139 is functionally relevant in regulating mammalian behavior.

## Significance Statement

GPR139 has been identified as a receptor with high expression in a key brain region for addiction, the habenula. However, no studies have yet provided causal evidence of the role of GPR139 in brain dysfunction. This study found that GPR139 receptor activation reduced alcohol self-administration in alcohol-dependent rats with compulsive-like drinking and decreased withdrawal-induced hyperalgesia. Importantly, we found that the habenula but not interpeduncular nucleus (IPN) was a mediator of the observed effects. These results represent an important advance in the field because they are the first to demonstrate a role for GPR139 in addiction-related behaviors.

## Introduction

Alcohol use disorder is a chronic relapsing disorder that is characterized by repeated cycles of excessive alcohol use and withdrawal. Major issues in the alcohol field include the limited number of medications that are available for the treatment of alcohol dependence and the lack of novel druggable targets beyond those that are associated with classic neurotransmitter systems (Volkow and Li, 2005). Currently, three medications have received United States Food and Drug Administration (FDA) approval for alcohol dependence (disulfiram, naltrexone, and acamprosate), but these drugs are associated with low compliance, modest effect sizes, and a return to excessive drinking when treatment is discontinued (Peterson, 2007; Garbutt, 2009; Olive, 2010). Therefore, it is critical to develop novel pharmacotherapies with larger effect sizes and fewer side effects to improve the treatment of alcohol use disorder.

G protein-coupled receptors (GPCRs) play an important role in mental disorders. A large proportion (30–60%) of current pharmaceutical drugs exert their therapeutic effects by targeting GPCRs (McCusker et al., 2007; Heng et al., 2013). Considering the prevalence and functional importance of GPCRs, unsurprising is that they remain among the most investigated targets for pharmaceutical discovery, especially because the current GPCR-targeting drugs affect only a small proportion of known GPCRs.

Orphan GPCRs are receptors for which endogenous ligands have not yet been fully identified. Most known GPCRs start as orphan GPCRs, and discoveries in the field have had a profound impact on our understanding of brain function (Civelli, 2012). Orphan GPCRs represent compelling novel targets in drug discovery. One such orphan GPCR, GPR139 (also known as GPRg1 or GPCR12), was first identified as a rhodopsin family GPCR that is almost exclusively expressed in the central nervous system (Gloriam et al., 2005; Matsuo et al., 2005). GPR139 receptors were also found to be highly conserved across species (Gloriam et al., 2005). The remarkably high sequence homology across different species and predominant brain expression suggest that GPR139 may play a critical role in brain function. Modulators of GPR139 receptor function may thus be particularly interesting for drug development for the treatment of mental disorders.

Interestingly, the highest expression of GPR139 receptors has been reported in the habenula (Matsuo et al., 2005; Wang et al., 2015; Hitchock et al., 2016), a brain region that has been shown to be critically involved in addiction, anxiety, and mood regulation (Fowler and Kenny, 2012; Batalla et al., 2017). A comparison of rodent and human transcriptome data recently identified a specific GPR139-including cluster of highly expressed habenular genes that are common to humans and rodents (Boulos et al., 2017). Interestingly, this cluster also expresses the μ opioid receptor, among others. In the medial habenula, GPR139-positive neurons project axons via the fasciculus retroflexus to the interpeduncular nucleus (IPN), where low GPR139 immunoreactivity is also detected (Liu et al., 2015). Both the habenula (Matsumoto and Hikosaka, 2007; Fowler et al., 2011; Hsu et al., 2014; Ishikawa and Kenny, 2016; Laurent et al., 2017) and IPN (Taraschenko et al., 2007; Antolin-Fontes et al., 2015; McLaughlin et al., 2017) have been identified as key regions in addiction.

Given the high expression of GPR139 in the habenula, we hypothesized that the modulation of GPR139 may be relevant to alcohol addiction-related behaviors. To test this hypothesis, we studied the effects of systemic administration of a selective GPR139 receptor agonist, JNJ-63533054, on alcohol self-administration in alcohol-nondependent and -dependent rats using chronic intermittent exposure (CIE) to alcohol vapor combined with operant self-administration. Addiction-related behaviors were assessed by measuring compulsive-like drinking despite adverse consequence (i.e., resistance to quinine adulteration), somatic signs of withdrawal, and mechanical nociception during withdrawal (i.e., withdrawal-induced hyperalgesia) in alcohol-dependent rats. We selected the CIE model because it has been shown to have predictive validity for alcohol use disorder in humans (Macey et al., 1996; Heilig and Koob, 2007). Finally, we tested the effects of intra-habenular and intra-IPN microinjections of JNJ-63553054 on alcohol self-administration and alcohol withdrawal-induced hyperalgesia in alcohol-dependent rats.

## Materials and Methods

### Subjects

Adult male Wistar rats (*n* = 46, Charles River), weighing 250–300 g at the beginning of the experiments, were used. The rats were group-housed, two per cage, in a temperature-controlled (22°C) animal facility on a 12/12 h light/dark cycle (lights off at 10 A.M.). The rats had access to food and water *ad libitum*. All animal procedures were conducted in adherence to the National Institutes of Health’s Guide for the Care and Use of Laboratory Animals and approved by The Scripps Research Institute Institutional Animal Care and Use Committee. All behavioral testing was conducted during the dark phase.

### Drugs

A 10% (v/v) alcohol drinking solution was prepared by diluting 95% alcohol with tap water. A 0.04% (w/v) saccharin drinking solution was prepared by diluting saccharin with tap water. JNJ-63533054 was synthesized at Janssen Research & Development as described previously (Dvorak et al., 2015; Liu et al., 2015). For systemic infusion, JNJ-63533054 was dissolved in 0.5% hydroxypropyl methylcellulose (HPMC) and administered orally 6 h into withdrawal, 60 min before behavioral testing, at doses of 10 and 30 mg/kg (2 ml/kg). The rats were habituated to the gastric gavage before starting the experiments. For intra-habenular and intra-IPN administration, JNJ-63533054 (0.25 µg/0.5 µl) was dissolved in vehicle that was composed of 5% dimethylsulfoxide, 5% emulphor, and 90% distilled water and infused 15 min before behavioral testing. For all of the self-administration studies, JNJ-63533054 was administered using a within-subjects design, and the order of injections of vehicle and JNJ-63533054 was counterbalanced. Quinine hydrochloride was purchased from Sigma-Aldrich. Increasing concentrations of quinine (0.005, 0.01, 0.025, and 0.05 g/l) were used to adulterate the alcohol drinking solution.

### Operant alcohol and saccharin self-administration

**Figure 1.**
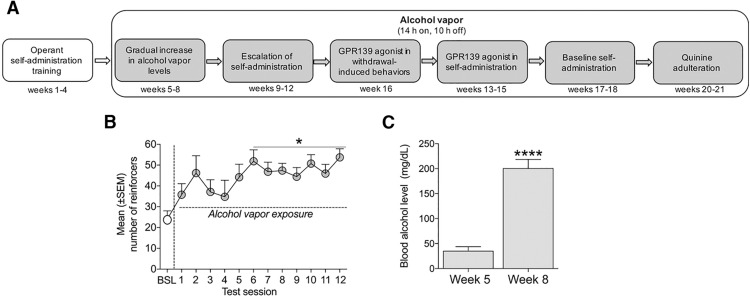
Timeline of the experiment, escalation of alcohol self-administration, and Blood alcohol levels: BALs after exposure to alcohol vapor. ***A***, Timeline of the experiment with systemic JNJ-63533054 administration in alcohol-dependent rats. ***B***, Rats that were exposed to alcohol vapor escalated their alcohol intake after the 6th session of operant self-administration; *p < 0.05, versus pre-vapor baseline. ***C***, BALs significantly increased after eight weeks of alcohol vapor exposure; ****p < 0.0001.

The rats were trained to self-administer 10% (v/v) alcohol solution during daily 30-min sessions in standard operant conditioning chambers (Med Associates) until stable responding was maintained (± 10% over the last three sessions). The rats were first subjected to an overnight session (12 h) in the operant chambers with access to one lever (right lever) that delivered water on a fixed-ratio 1 (FR1) schedule of reinforcement (i.e., each operant response was reinforced with 0.1 ml of water). After 1 d off, the rats were subjected to a 3-h session (FR1) with access to the right lever that delivered 0.1 ml of alcohol. In the next two sessions, the rats were subjected to 2-h and 1-h FR1 sessions, respectively, with the right lever delivering 0.1 ml of alcohol. After this training phase, all subsequent sessions lasted 30 min, with both levers available for water and alcohol (left lever for water and right lever for alcohol) until stable levels of intake were reached. For the saccharin self-administration study, the rats underwent daily 30 min of FR1 sessions. Responses on the right lever resulted in the delivery of 0.1 ml of saccharin (0.04%, w/v). Lever presses on the left lever delivered 0.1 ml of water. This training lasted two weeks until a stable baseline of intake was reached. Behavioral testing occurred three times per week during the dark phase (for the experimental design, see Figs. 1*A*, 6*A*).

### Mechanical nociceptive von Frey test during alcohol withdrawal

Mechanical nociception, reflected by hindpaw withdrawal thresholds, was determined by an observer who was blind to the experimental condition using von Frey filaments, ranging from 8.511 to 281.838 g. JNJ-63533054 was administered orally in a between-subjects design (*n* = 8–9) 60-min before starting the experiment. A test session began after 10 min of habituation to the testing environment. A series of von Frey filaments was applied from below the wire mesh to the central region of the plantar surface of the left hindpaw in ascending order, beginning with the smallest filament (8.511 g). The filament was applied until buckling of the hair occurred, and the filament remained in place for ∼2 s. Rapid withdrawal of the hindpaw was considered a positive response. The stimulus was incrementally increased until a positive response was observed and then decreased until a negative response was observed to determine a pattern of responses to apply to the statistical methods that were described by Dixon (1980). Once the threshold was determined for the left hindpaw, the same testing procedure was applied to the right hindpaw after 5 min. The 50% paw withdrawal threshold was calculated as *Xf + kδ*, where *Xf* is the last von Frey filament employed, *k* is the Dixon value that corresponds to the response pattern, and *δ* is the mean difference between stimuli. Paw withdrawal thresholds were determined for alcohol-dependent rats during withdrawal.

### Somatic withdrawal score

Behavioral signs of withdrawal were measured using a rating scale that was adapted from previous studies (Macey et al., 1996; de Guglielmo et al., 2017). JNJ-63533054 was administered orally in a between-subjects design (*n* = 8–9) 60-min before beginning the experiment. The observer was blind to the experimental condition. The following signs were measured: ventromedial limb retraction, irritability to touch (vocalization), tail rigidity, abnormal gait, and body tremors. Each sign was given a score of 0–2, based on the following severity scale: 0 = no sign, 1 = moderate, and 2 = severe. The sum of the four observation scores (0–8) was used as an operational measure of withdrawal severity.

### Effect of systemic JNJ-63533054 administration on alcohol self-administration in nondependent and alcohol-dependent rats

Once a stable baseline of operant alcohol self-administration was reached, the rats were divided into two groups: alcohol-dependent (*n* = 17) and nondependent (*n* = 12). A separate cohort of rats was trained to self-administer saccharin (0.04%, w/v) and made alcohol-dependent after stable operant saccharin self-administration was reached. The rats in the alcohol-dependent groups were made dependent by CIE to alcohol vapor as described previously (O'Dell et al., 2004; Ron and Barak, 2016). Briefly, the rats underwent cycles of 14 h of alcohol vapor ON and 10 h of alcohol vapor OFF in their home cages for four consecutive weeks with no operant self-administration. Tail blood was collected during vapor exposure and used to determine blood alcohol levels (BALs) using gas chromatography. Vapor exposure was gradually increased until BALs ranged from 150 to 250 mg/dl. After four weeks of vapor exposure, the rats resumed operant self-administration sessions for four weeks to allow them to escalate their alcohol intake. Rats that are made dependent with CIE exhibit clinically relevant BALs, ranging from 150 to 250 mg/dl during vapor exposure (Gilpin et al., 2009), compulsive-like alcohol drinking (i.e., persistent drinking despite aversive, bitter taste of quinine; Vendruscolo et al., 2012; Seif et al., 2013; Leão et al., 2015; Kimbrough et al., 2017a), and the escalation of alcohol drinking when given access to alcohol during abstinence (O'Dell et al., 2004; Kimbrough et al., 2017a). Additionally, alcohol dependence that is induced by alcohol vapor results in both somatic and motivational withdrawal symptoms during both acute withdrawal and protracted abstinence, including anxiety-like behavior, irritability-like behavior, and mechanical hyperalgesia (Macey et al., 1996; Valdez et al., 2002; Edwards et al., 2012; Williams et al., 2012; Kallupi et al., 2014; Vendruscolo and Roberts, 2014; de Guglielmo et al., 2016, 2017; Kimbrough et al., 2017b; Smith et al., 2017). Behavioral testing following systemic JNJ-63533054 administration occurred during acute withdrawal (6–8 h after alcohol vapor was turned OFF), during which both brain and BALs are negligible (Gilpin et al., 2009). Rats that received intracerebral infusions of JNJ-63533054 underwent surgery after stable, escalated alcohol intake was reached. Alcohol-dependent rats were maintained on CIE until the end of the experiment.

### Quinine adulteration of alcohol

After behavioral testing, the rats were maintained on an FR1 schedule of operant self-administration until stable levels of alcohol self-administration were established again. To further test for compulsive-like alcohol drinking, quinine adulteration was used, which has been validated as a measure of compulsive-like responding for alcohol (Vendruscolo et al., 2012; Seif et al., 2013; Leão et al., 2015; Kimbrough et al., 2017a). This test measures the persistence of animals to consume alcohol despite the aversive bitter taste of quinine. The alcohol solution was adulterated with increasing concentrations of quinine (0.005, 0.01, 0.025, and 0.05 g/l) and presented between operant self-administration sessions (one concentration per session). To test the selectivity of responses for adulterated alcohol, water intake was assessed using the quinine concentration (0.025 g/l) at which a significant difference in alcohol intake was observed between the subgroups of rats.

### Intracerebral surgery and effect of site-specific microinjections of JNJ-63533054 on alcohol self-administration in dependent rats

The rats were made dependent on alcohol using the aforementioned protocol and then implanted with intracerebral cannulae that targeted the medial habenula and IPN. The coordinates were based on a previous study (Tuesta et al., 2017) and modified slightly based on trial infusions. For intracerebral surgery, the animals were anesthetized with isoflurane (5% induction, 1.5–2.5% maintenance). To reach the medial habenula, 24-gauge guide cannulae (Plastics One) were implanted bilaterally using the following coordinates with reference to bregma: 10° angle toward midline; anterior/posterior: -3.2 mm; medial/lateral: ±1.35 mm, dorsal/ventral: -3.3 mm from dura. For the IPN, the coordinates were the following: 10° angle toward midline; anterior/posterior: -6.72 mm; medial/lateral: ±1.6 mm; dorsal/ventral: -6.5 mm from dura. During the injections, the 31-gauge injector needles extended 2 mm below the tip of the cannula for placement into the targeted brain region. The animals were allowed to recover for one week after surgery and thereafter returned to the alcohol vapor chambers and allowed to self-administer alcohol until the baseline level of alcohol intake pre-surgery was restored. The rats were then treated with either vehicle or JNJ-63533054 (0.25 μg/0.5 μl) according to a within-subjects Latin-square design (counterbalanced for order). For the microinjections, an infusion volume of 0.5 μl was delivered over 2 min using a Hamilton syringe (Hamilton) that was attached to an infusion pump (kdScientific) via 31-gauge injectors that projected 2 mm beyond the tip of the cannulae. The injectors were left in place for 1 min following the infusion to allow for diffusion and reduce backflow. Cannula placements were determined according to the Paxinos and Watson rat brain atlas (Paxinos and Watson, 2007). The histologic verification of cannula placements revealed three misplacements (two for the medial habenula and one for the IPN); the data from these rats were excluded, leaving six rats for habenular infusions and seven rats for IPN infusions for the final analysis.

### Experimental design and statistical analysis

The effects of systemic JNJ-63533054 administration on alcohol self-administration were assessed in both nondependent (*n* = 12) and alcohol-dependent (*n* = 17) male rats using a within-subjects design. To study the effect of JNJ-6353054 on operant self-administration of a natural reinforcer, saccharin, a separate cohort was made alcohol dependent (*n* = 9) and trained to self-administer saccharin during alcohol withdrawal. The data were analyzed using one-way repeated-measures ANOVAs followed by the Newman–Keuls multiple-comparison *post hoc* test. The effects of systemic JNJ-63533054 administration on mechanical nociception and somatic signs of withdrawal in alcohol-dependent rats were then analyzed during alcohol withdrawal using a between-subjects design by comparing the vehicle-treated control group (*n* = 8) with the JNJ-63533054-treated group (*n* = 9). The data were analyzed using unpaired two-tailed Student’s *t* test (for mechanical nociception) or the nonparametric Mann–Whitney test (for somatic withdrawal signs). Quinine-adulterated alcohol intake was assessed in alcohol-dependent rats, in which each rat received gradual increases in quinine-adulterated alcohol solution. The data were analyzed using two-way repeated-measures ANOVA followed by the Newman–Keuls multiple-comparison *post hoc* test. At the end of the quinine experiment, quinine-adulterated water intake was analyzed using unpaired two-tailed Student’s *t* test. Based on compulsive-like alcohol drinking, the effect of JNJ-63533054 in alcohol-dependent rats was analyzed using repeated-measures two-way ANOVA followed by the Newman–Keuls *post hoc* test. Cohen’s *d* was used to calculate the effect size of JNJ-63533054 on paw withdrawal thresholds separately in low-compulsive and high-compulsive rats. A separate cohort of alcohol-dependent rats (*n* = 8) was implanted with cannulae in both the medial habenula and IPN. The effects of intracerebral infusions of JNJ-63533054 on self-administration and mechanical nociception were assessed using a within-subjects design. Data from both experiments that intracerebrally infused JNJ-63533054 (*n* = 6 for habenula and *n* = 7 for IPN) were analyzed using repeated-measures one-way ANOVA followed by the Newman–Keuls multiple-comparison *post hoc* test. The statistical analyses were performed using Statistica and GraphPad Prism 7 software. The statistical analyses, data structure, and results are presented in Table 1.

**Table 1.** Statistical table

Figure	Data structure	Type of test	Statistical value	*p*
1*B*	One factor (time)	One-way repeated-measures ANOVA	*F*_(12,192)_ = 2.618	0.0030
		Newman–Keuls		<0.05
1*C*	Normal distribution, two-tailed	Paired *t* test	*t*_(16)_ = 7.301	<0.0001
2*A*	One factor (treatment)	One-way repeated-measures ANOVA	*F*_(4,64)_ = 16.92	<0.0001
		Newman–Keuls		<0.01
2*C*	One factor (treatment)	One-way repeated-measures ANOVA	*F*_(3,33)_ = 2.16	0.1114
2*D*	One factor (treatment)	One-way repeated-measures ANOVA	*F*_(3.24)_ = 0.1766	0.9112
3*A*	Normal distribution, two-tailed	Unpaired *t* test	*t*_(15)_ = 2.943	0.0101
3*B*	Nonparametric	Mann–Whitney *U*	*U* = 25.5	0.3214
4*A*	Normal distribution, two-tailed	Unpaired *t* test	*t*_(15)_ = 4.908	0.0002
4*B*	Two factors (compulsivity, treatment)	Two-way repeated-measures ANOVA	Interaction: *F*_(4,60)_ = 3.254	0.0174
		Newman–Keuls		<0.05 (0.005 g/l for low-compulsive rats)>0.05 (0.005 g/l for high-compulsive rats)
4*C*	Normal distribution, two-tailed	Unpaired *t* test	*t*_(15)_ = 0.4353	0.6695
5*A*	Two factors (compulsivity, treatment)	Two-way repeated-measures ANOVA	Interaction: *F*_(4,60)_ = 3.191	0.0192
		Newman–Keuls		>0.05 (30 mg/kg for low-compulsive rats)<0.01 (30 mg/kg for high-compulsive rats)
5*B*	Normal distribution, two-tailed	Paired *t* test	*t*_(8)_ = 5.357	0.0007
6*B*	One factor (treatment)	One-way repeated-measures ANOVA	*F*_(3,15)_ = 11.71	0.0003
		Newman–Keuls		<0.01
6*C*	Normal distribution, two-tailed	Paired *t* test	*t*_(5)_ = 5.709	0.0023
6*F*	One factor (treatment)	One-way repeated-measures ANOVA	*F*_(3,18)_ = 7.459	0.0019
		Newman–Keuls		>0.05
6*G*	Normal distribution, two-tailed	Paired *t* test	*t*_(6)_ = 0.1455	0.8891

## Results

### Systemic administration of the GPR139 receptor agonist JNJ-63533054 selectively decreases alcohol self-administration without affecting saccharin self-administration in alcohol-dependent rats

The timeline of the experiment with systemic JNJ-63533054 administration in alcohol-dependent rats is shown in Figure 1*A*. The dependent animals significantly escalated their alcohol intake after two weeks, starting in the 6th test session (Fig. 1*B*). This was confirmed by one-way repeated-measures ANOVA (*F*_(12,192)_ = 2.618, *p* = 0.0030) followed by the Newman–Keuls *post hoc* test (*p* < 0.05). There was a significant increase in BALs from week 5 to week 8 of alcohol exposure, confirmed by the paired *t* test (*t*_(16)_ = 7.301, *p* < 0.0001; Fig. 1*C*).

**Figure 2.**
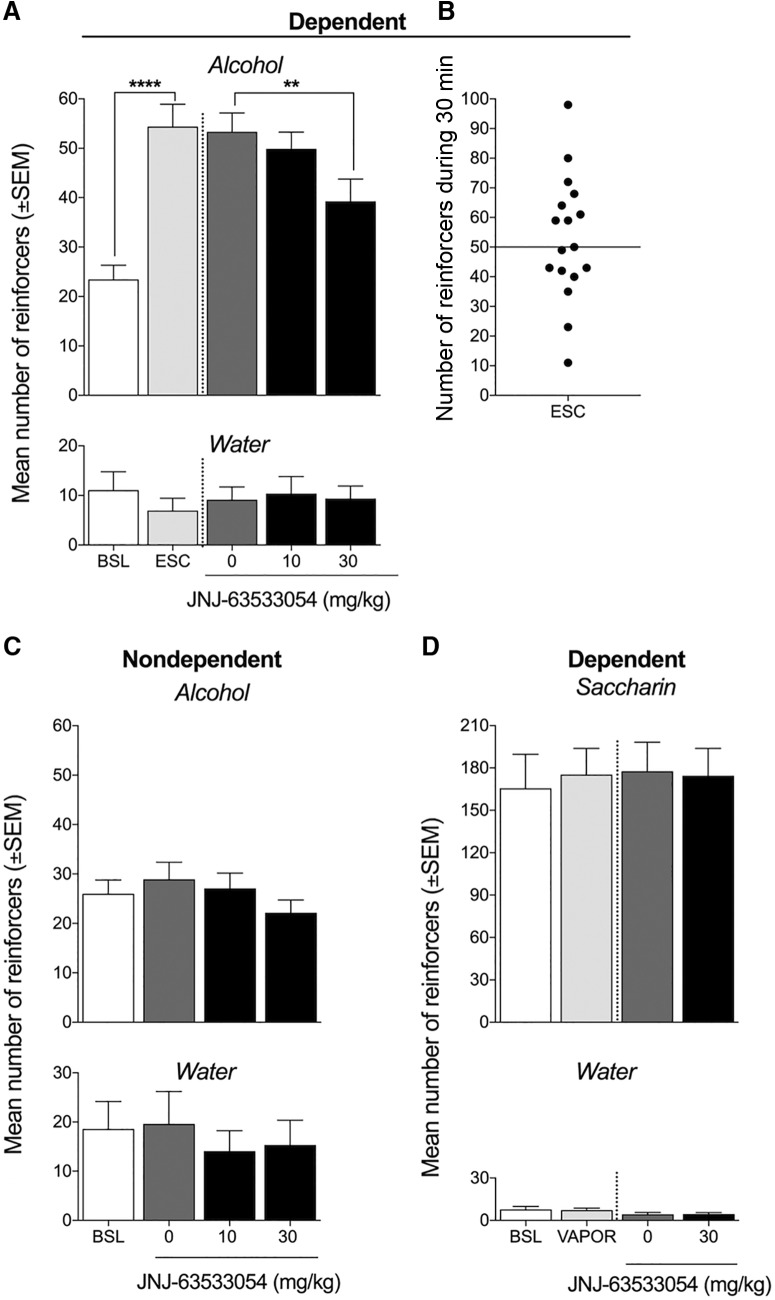
GPR139 receptor agonist JNJ-63533054 reverses the escalation of alcohol self-administration in alcohol-dependent rats, with no effect in nondependent rats. ***A***, Rats in the alcohol-dependent group were made dependent by chronic intermittent alcohol vapor exposure. Once the animals were made alcohol-dependent and escalated their alcohol intake [****p < 0.0001, pre-vapor baseline (BSL) vs escalated baseline (ESC)], the effect of JNJ-63533054 on alcohol self-administration was evaluated using a within-subjects design (*n* = 17). One hour before the session, the rats were orally administered a single dose of JNJ-63533054. JNJ-63533054 significantly reduced alcohol self-administration at a dose of 30 mg/kg (**p < 0.05). Water intake was unaffected by JNJ-63533054 treatment. ***B***, The median number of reinforced responses for alcohol in alcohol-dependent rats was 50 after escalation. ***C***, Once a stable baseline of alcohol intake was reached (±10% over the last three sessions), the effect of JNJ-63533054 on alcohol intake was tested in nondependent rats. One hour before the session, the rats were orally administered a single dose of JNJ-63533054 in a within-subjects design (*n* = 12). JNJ-63533054 did not significantly affect alcohol self-administration in nondependent rats. Water self-administration was unaffected by JNJ-63533054 treatment. ***D***, The effect of JNJ-63533054 (30 mg/kg, p.o.) on 0.04% (w/v) saccharin self-administration was tested in a separate cohort of rats that were made alcohol dependent by chronic intermittent alcohol vapor exposure. Both saccharin and water self-administration was unaffected by vapor exposure or JNJ-63533054.

Once the alcohol-dependent rats reached a stable level of operant responding for alcohol, a within-subjects design (*n* = 17; Fig. 2*A*) was used to evaluate the effects of JNJ-63533054 on alcohol self-administration. The one-way repeated-measures ANOVA revealed a significant effect of systemic JNJ-63533054 administration on alcohol self-administration (*F*_(4,64)_ = 16.92, *p* < 0.0001). The Newman–Keuls *post hoc* test showed that JNJ-63533054 significantly reduced alcohol self-administration at the dose of 30 mg/kg (*p* < 0.01), with no effect on water intake at either dose of JNJ-63533054 (*F*_(4,64)_ = 0.3664, *p* = 0.8317; Fig. 2*A*). The median number of reinforcers per 30-min self-administration session after escalation was 50 reinforcers (Fig. 2*B*). JNJ-63533054 did not affect alcohol self-administration in nondependent rats, confirmed by the one-way repeated-measures ANOVA (*F*_(3,33)_ = 2.16, *p* = 0.1114; Fig. 2*C*). Water self-administration was unaffected by JNJ-63533054 treatment (*F*_(3,33)_ = 1.54, *p* = 0.2225) in nondependent rats. A separate cohort of rats was made alcohol dependent, in which exposure to alcohol vapor or JNJ-63533054 (30 mg/kg) had no effect on saccharin self-administration (*F*_(3.24)_ = 0.1766, *p* = 0.9112; Fig. 2*D*) or water self-administration (*F*_(3.24)_ = 2.405, *p* = 0.0923), confirmed by the one-way repeated-measures ANOVA.

### Systemic administration of the GPR139 receptor agonist JNJ-63533054 increases pain thresholds during alcohol withdrawal, without affecting somatic signs of withdrawal

**Figure 3.**
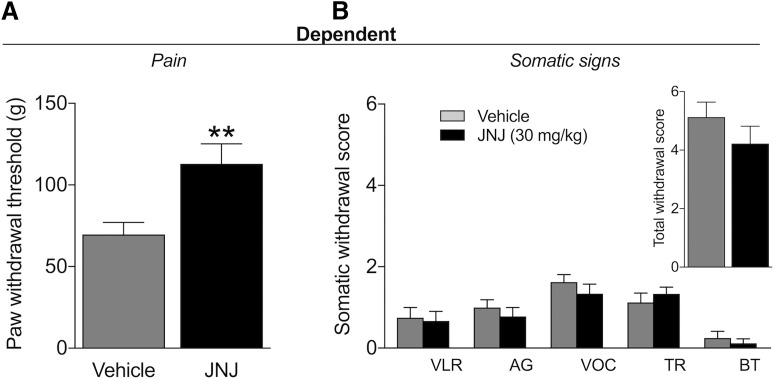
GPR139 agonist decreases withdrawal-induced hyperalgesia without affecting somatic signs of withdrawal. ***A***, JNJ-63533054 (30 mg/kg, p.o.) increased paw withdrawal thresholds compared with vehicle-treated rats in the mechanical nociceptive von Frey test (**p < 0.05), indicating an increase in pain thresholds during alcohol withdrawal. ***B***, JNJ-63533054 did not affect the number of somatic signs of alcohol withdrawal; *n* = 8–9/group. VLR: ventromedial limb retraction, VOC: vocalization, TR: tail rigidity, AG: abnormal gait, and BT: body tremors.

Mechanical hyperalgesia and somatic signs of withdrawal were evaluated 7-8 h into withdrawal using a between-subjects design (*n* = 8–9). The results showed a higher threshold for mechanical nociception in JNJ-63533054-treated rats (Fig. 3*A*). This was confirmed by the unpaired *t* test (*t*_(15)_ = 2.943, *p* = 0.0101). No significant differences in somatic withdrawal signs were found between the two groups (Fig. 3*B*), confirmed by the nonparametric Mann–Whitney *U* test (*U* = 25.5, *p* = 0.3214).

### Alcohol-dependent rats with higher baseline alcohol intake exhibit resistance to quinine adulteration

**Figure 4.**
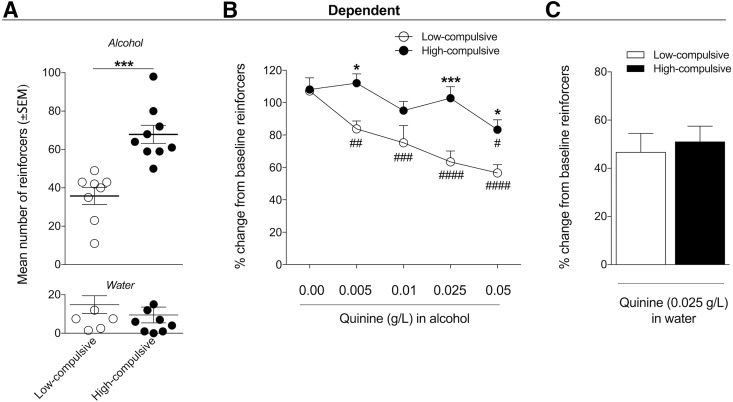
Dependent rats that exhibit high alcohol drinking exhibit high compulsive-like alcohol drinking. ***A***, Two distinct subgroups of rats in the alcohol-dependent group were identified according to the median of 50 alcohol-reinforced responses during baseline alcohol self-administration after escalation. Baseline alcohol intake was significantly higher in high-compulsive rats compared with low-compulsive rats (***p < 0.001, *n* = 8–9). ***B***, To further test the compulsivity of alcohol intake, the rats in the low- and high-compulsive subgroups underwent the quinine adulteration test. The rats were subjected to operant alcohol self-administration sessions with increasing concentrations of quinine that were added to the alcohol solution. The data are expressed as the percentage change relative to the escalated baseline (i.e., lever presses for alcohol alone before quinine adulteration). High-compulsive rats maintained their alcohol drinking despite the aversive, bitter taste of quinine in the alcohol solution (i.e., they were high-compulsive alcohol drinkers). Low-compulsive rats decreased their alcohol intake (>20% from baseline) starting with the lowest concentration of quinine (0.005 g/l; i.e., they were low-compulsive), whereas only the highest quinine concentration (0.05 g/l) decreased alcohol intake in high-compulsive rats; #*p* < 0.05, ##*p* < 0.01, ###*p* < 0.001, ####*p* < 0.0001, significant difference compared with own baseline; *p < 0.05, ***p < 0.001, significant difference between low-compulsive rats and high-compulsive rats. ***C***, The intake of quinine (0.025 g/l)-adulterated water was not different between low-compulsive and high-compulsive rats.

Based on the median reinforcers/session after escalation in dependent rats, two distinct subgroups of rats were identified: one with below-median baseline alcohol intake (<50 reinforcers/session, *n* = 8) and one with above-median baseline alcohol intake (>50 reinforcers/session, *n* = 9; Fig. 4*A*). This was confirmed by a significant difference in baseline alcohol intake between the two subgroups (*t*_(15)_ = 4.908, *p* = 0.0002). Water intake was not different between the two subgroups (*t*_(15)_ = 0.8706, *p* = 0.3977; Fig. 4*A*). No significant difference in body weight was found between the two subgroups (data not shown), indicating that the number of lever presses and consequently alcohol intake were independent of body weight. To further analyze compulsive-like responding for alcohol in these two subgroups, we used the quinine adulteration test, which measures the persistence of alcohol drinking despite the aversive bitter taste of quinine. The subgroups of rats with low baseline alcohol intake also exhibited low compulsive-like drinking, indicated by 10-fold higher sensitivity to quinine (0.005 g/l) compared with high-compulsive rats (0.05 g/l). Figure 4*B* shows the average number of rewards during the quinine adulteration test with each increasing concentration of quinine between low-intake (i.e., low-compulsive) and high-intake (i.e., high-compulsive) rats (*n* = 8 low-compulsive rats, *n* = 9 high-compulsive rats). The two-way repeated-measures ANOVA revealed a significant time × group interaction (*F*_(4,60)_ = 3.254, *p* = 0.0174) and significant effects of quinine concentration (*F*_(4,60)_ = 12.31, *p* < 0.0001) and group (*F*_(1,15)_ = 20.29, *p* = 0.0004). The Newman–Keuls multiple-comparison test confirmed a significant difference between the subgroups of rats at quinine concentrations of 0.005 g/l (*p* < 0.05), 0.025 g/l (*p* < 0.01), and 0.05 g/l (*p* < 0.05). The Newman–Keuls multiple-comparison *post hoc* test indicated that quinine adulteration decreased lever pressing in the low-compulsive group beginning at the lowest concentration of quinine (0.005 g/l) compared with baseline (*p* < 0.05 for 0.05 g/l, *p* < 0.001 for 0.01 g/l, *p* < 0.0001 for 0.05 g/l), whereas only the highest concentration of quinine (0.05 g/l) decreased lever-pressing in the high-compulsive group (*p* < 0.05). To verify that the effect of quinine was selective for alcohol and did not merely indicate a difference in taste sensitivity between low-compulsive and high-compulsive rats, we tested the quinine concentration (0.025 g/l) that caused the most significant difference in alcohol intake between groups (*p* < 0.01) on quinine-adulterated water intake between groups (Fig. 4*C*). The *t* test indicated no significant difference in lever-pressing for quinine-adulterated water (*t*_(15)_ = 0.4353, *p* = 0.6695).

### JNJ-63533054 decreases alcohol self-administration only in a subgroup of high-intake rats that exhibit compulsive-like drinking

**Figure 5.**
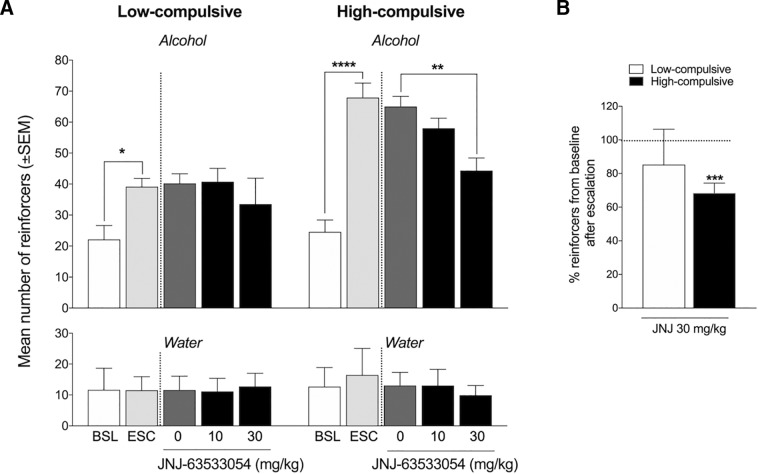
JNJ-63533054 decreases alcohol intake in high-compulsive alcohol-dependent rats but has no effect on alcohol intake in low-compulsive alcohol-dependent rats. ***A***, Rats in the high-compulsive subgroup of alcohol-dependent rats (*n* = 9) escalated their alcohol intake [****p < 0.0001, pre-vapor baseline (BSL) vs escalated baseline (ESC)]. JNJ-63533054 (30 mg/kg, p.o.) significantly decreased alcohol intake in high-compulsive rats (**p < 0.01). Rats in the low-compulsive subgroup escalated their alcohol intake after alcohol vapor exposure (*p < 0.05), but JNJ-63533054 had no effect on alcohol intake in low-compulsive rats (*n* = 8). ***B***, JNJ-63533054 (30 mg/kg) decreased (>30% reduction) alcohol self-administration in high-compulsive rats (***p < 0.0001) but had no effect in low-compulsive rats.

Dependent rats were divided into two subgroups according to both the baseline escalated number of self-administered reinforcers [below the median for low-compulsive rats and above the median for high-compulsive rats (50)] and compulsive-like alcohol consumption in the quinine adulteration test, with a <20% reduction of alcohol intake at the lowest concentration of quinine (0.005 g/l) in low-compulsive rats (i.e., 10-fold higher sensitivity to 0.005 g/l quinine) compared with high-compulsive rats. High-compulsive rats (*p* = 0.00014; Fig. 5*A*) and low-compulsive rats (*p* = 0.0287) escalated their alcohol intake from pre-vapor baseline to post-vapor baseline (i.e., escalation), confirmed by the two-way repeated-measures ANOVA (subgroup × treatment interaction, *F*_4,60_ = 3.191, *p* = 0.0192) followed by the Newman–Keuls *post hoc* test. The Newman–Keuls *post hoc* test showed that JNJ-63533054 significantly reduced alcohol self-administration at a dose of 30 mg/kg (*p* = 0.00274). JNJ-63533054 did not affect alcohol self-administration in low-compulsive rats at either dose tested (*p* < 0.05). The two-way repeated-measures ANOVA indicated no significant difference in water intake between pre-vapor and post-vapor and no effect of JNJ-63533054 treatment in either high-compulsive rats or low-compulsive rats, reflected by a lack of interaction (*F*_(4,60)_ = 0.2027, *p* = 0.9359). JNJ-63533054 (30 mg/kg) induced a 31.6 ± 5.9% (mean ± SEM) reduction of intake compared with baseline in high-compulsive rats (*t*_(8)_ = 5.357, *p* = 0.0007; Fig. 5*B*), whereas no significant reduction was observed in low-compulsive rats (14.7 ± 21.1%, *t*_(7)_ = 0.6963, *p* = 0.5087).

Mechanical hyperalgesia was also analyzed in the different subgroups (*n* = 3-5/group, data not shown). Pretreatment with JNJ-63533054 caused a 121.18 ± 8.11% increase in paw withdrawal thresholds in low-compulsive rats and 174.39 ± 42.06% increase in high-compulsive rats. The analysis of the effect size showed that JNJ-63533054 treatment had a greater effect in high-compulsive rats (Cohen’s *d* = 0.7593 for low-compulsive rats and 1.1740 for high-compulsive rats).

### Intra-habenular but not intra-IPN infusion of JNJ-63533054 decreases alcohol self-administration and mechanical hyperalgesia in alcohol-dependent rats

**Figure 6.**
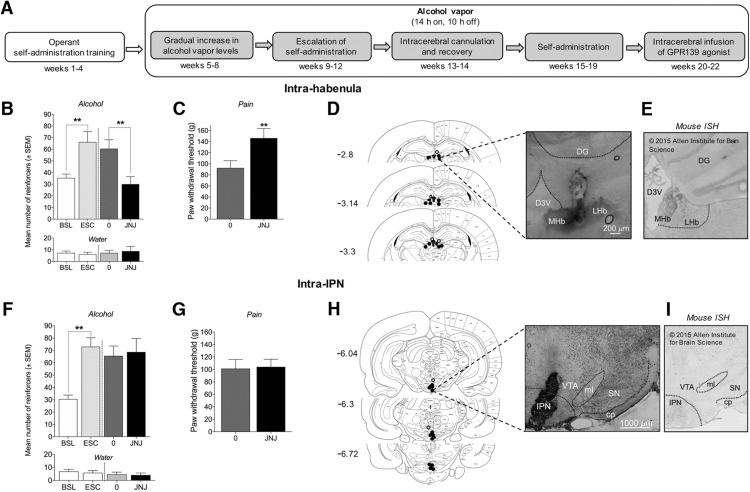
Intra-habenular but not intra-IPN JNJ-63533054 administration decreases alcohol intake and increases paw withdrawal thresholds in alcohol-dependent rats during withdrawal. ***A***, Timeline of microinfusions of JNJ-63533054 in alcohol-dependent rats. ***B***, Intra-habenular infusion of JNJ-63533054 (0.25 µg/0.5 µl) decreased alcohol self-administration in dependent rats (**p < 0.01), without affecting water self-administration (*n* = 6). ***C***, Intra-habenular infusion of JNJ-63533054 increased paw withdrawal thresholds during alcohol withdrawal (**p < 0.01). ***D***, Histology of accurate injection sites in the habenula (black circles) and misplaced injection sites (white circles); 5× magnification. ***E***, *In situ* hybridization of GPR139 receptors in mouse habenula. Modified from Allen Mouse Brain Atlas (AllenMouseBrainAtlas, 2004). ***F***, Intra-IPN infusion of JNJ-63533054 did not affect alcohol or water self-administration in alcohol-dependent rats (*n* = 7). ***G***, Paw withdrawal thresholds during alcohol withdrawal were unaffected by intra-IPN infusion of JNJ-63533054. ***H***, Histology of accurate injection sites in the IPN (black circles) and misplaced injection sites (white circles); 2.5× magnification. ***I***, *In situ* hybridization of GPR139 receptors in the mouse IPN. Modified from Allen Mouse Brain Atlas (AllenMouseBrainAtlas, 2004). cp, cerebral peduncle; DG, dentate gyrus; D3V, dorsal third ventricle; LHb, lateral habenula; MHb, medial habenula; ml, medial lemniscus; SN, substantia nigra; VTA, ventral tegmental area.

To identify the brain circuits that mediate the effects of JNJ-63533054 on alcohol self-administration and withdrawal-induced mechanical hyperalgesia, a separate cohort of alcohol-dependent rats was implanted with intra-habenular and intra-IPN cannulae. The timeline for intracerebral infusions of JNJ-63533054 is presented in Figure 6*A*. In the intra-habenular group, rats that received CIE to alcohol vapor escalated their alcohol intake from baseline, confirmed by one-way repeated-measures ANOVA (*F*_(3,15)_ = 11.71, *p* = 0.0003) followed by the Newman–Keuls *post hoc* test (baseline versus escalation, *p* < 0.01; Fig. 6*B*). The intra-habenular infusion of JNJ-63533054 (0.25 µg/0.5 µl) significantly decreased alcohol self-administration, confirmed by the Newman–Keuls *post hoc* test (*p* < 0.01), without affecting water self-administration (*F*_(3,15)_ = 0.3253, *p* = 0.8071; Fig. 6*B*). The intra-habenular infusion of JNJ-63533054 also increased paw withdrawal thresholds, indicating a decrease in hyperalgesia during alcohol withdrawal (*t*_(5)_ = 5.709, *p* = 0.0023; Fig. 6*C*). In the intra-IPN group, rats that received CIE to alcohol vapor escalated their alcohol intake from baseline, confirmed by the one-way repeated-measures ANOVA (*F*_(3,18)_ = 7.459, *p* = 0.0019) followed by the Newman–Keuls *post hoc* test (baseline versus escalation, *p* < 0.01; Fig. 6*F*). The intra-IPN infusion of JNJ-63533054 did not affect alcohol self-administration (*p* > 0.05; Fig. 6*F*) or paw withdrawal thresholds (*t*_(6)_ = 0.1455, *p* = 0.8891; Fig. 6*G*). At the end of the experiments, cannula placements in the habenula (Fig. 6*D*) and IPN (Fig. 6*H*) were verified.

## Discussion

The present study showed that GPR139 receptor agonism decreases alcohol intake selectively in alcohol-dependent rats, without affecting saccharin intake in alcohol-dependent rats or alcohol intake in nondependent rats. Additionally, the reduction of alcohol intake was observed in a subgroup of alcohol-dependent rats that exhibited a compulsive intake-like phenotype. JNJ-63533054 had no effect on somatic withdrawal signs during acute withdrawal, but a decrease in withdrawal-induced mechanical hyperalgesia was observed after systemic JNJ-63533054 administration. Importantly, the intra-habenular but not intra-IPN infusion of JNJ-63533054 decreased both alcohol self-administration and withdrawal-induced hyperalgesia in alcohol-dependent rats. Overall, we found that GPR139 receptor activation specifically in the habenula selectively reduced key addiction-like behaviors in an advanced preclinical model of alcohol use disorder.

These results demonstrate that GPR139 activation is only effective in alcohol-dependent rats, without affecting alcohol intake in nondependent rats. Additionally, JNJ-63533054 had no effect on saccharin intake in alcohol-dependent rats. The selective effect of JNJ-63533054 on alcohol intake suggests that GPR139 may be involved in functional processes that underlie drug dependence and addiction (e.g., by altering the level of GPR139 expression in the brain) and not merely in the reduction of the rewarding effects of alcohol or saccharin.

The analysis of individual differences in compulsive-like alcohol drinking indicated that GPR139 receptor activation was particularly effective in alcohol-dependent animals that exhibited high, compulsive-like alcohol drinking. The quinine adulteration test has been previously validated as a model of compulsive-like drinking (Vendruscolo et al., 2012; Seif et al., 2013; Leão et al., 2015; Kimbrough et al., 2017a). In the model of CIE to alcohol vapor, abstinence from alcohol has been shown to trigger an aversive withdrawal syndrome, during which rats exhibit somatic and motivational signs, including hyperalgesia (Edwards et al., 2012; Vendruscolo and Roberts, 2014; de Guglielmo et al., 2017). Additionally, withdrawal from chronic alcohol exposure increases pain sensitivity in alcoholic-dependent humans (Jochum et al., 2010), and chronic intermittent voluntary alcohol intake has been shown to induce hyperalgesia during withdrawal in rats (Fu et al., 2015). Although JNJ-63533054 had no effect on somatic signs of withdrawal, it significantly decreased withdrawal-induced hyperalgesia, measured by paw-withdrawal thresholds in the von Frey test. The increase in paw-withdrawal thresholds was greater in high-compulsive rats compared with low-compulsive rats (174.39 ± 42.06% and 121.15 ± 8.11% increases, respectively). In the CIE model, withdrawal-induced hyperalgesia is usually associated with a 25–35% decrease in mechanical thresholds compared with naive or nondependent animals (Edwards et al., 2012; de Guglielmo et al., 2017). Therefore, we hypothesize that JNJ-63533054 treatment may have completely restored pain thresholds to normal levels. Future studies that employ longitudinal designs will be required to test this hypothesis.

Interestingly, lesions of the medial habenula have been shown to increase pain sensitivity and increase the analgesic effect of morphine (Mészáros et al., 1985). A recent study found that the analgesic effect of morphine is mediated by the medial habenula (Darcq et al., 2012). Given that the highest expression of GPR139 receptors is found in the medial habenula (Matsuo et al., 2005; Liu et al., 2015; Hitchock et al., 2016), GPR139 receptors may modulate opioidergic systems in the medial habenula to produce the analgesic effects that were observed in the present study. Indeed, a comparison of rodent and human transcriptome data revealed a specific GPR139-including cluster of highly expressed habenular genes that are common to humans and rodents that also notably express μ opioid receptors (Boulos et al., 2017). We observed no effect of JNJ-63533054 on somatic withdrawal signs. The GPR139-mediated reduction on withdrawal symptoms was selective to hyperalgesia, further indicating that GPR139 may interact with the opioidergic system in the habenula.

JNJ-63533054 is a selective, high-affinity, small-molecule agonist of GPR139 receptors that has good drug-like properties, including favorable pharmacokinetics and brain penetration after oral dosing in rats, with no cytochrome P450 (CYP450) inhibition (Dvorak et al., 2015). To our knowledge, only one behavioral study has been published to date regarding GPR139 receptors, in which the GPR139 receptor agonist JNJ-63533054 (10 and 30 mg/kg) induced spontaneous hypolocomotion during the first hour after the injection (Liu et al., 2015). To avoid the potential confound of hypolocomotion, we delayed the testing of alcohol self-administration for 1 h after systemic JNJ-6353054 administration. Considering that we did not observe any decrease in water drinking in any of the groups, did not observe decreases in alcohol drinking in nondependent rats, and did not observe a reduction of operant responding for the natural reinforcer saccharin, hypolocomotion unlikely explains the present results.

One limitation of the present study is the lack of GPR139 antagonist administration. The lack of availability of such antagonists is a known weakness in the field. Although several small-molecule antagonists have been shown to have reasonable potency *in vitro*, little to no selectivity data have been reported (Hu et al., 2009; Wang et al., 2015). Additionally, no studies of safety and efficacy *in vivo* have been reported. Whether these compounds are selective and brain-penetrant, have favorable pharmacokinetics, and are suitable for *in vivo* experiments remains to be investigated.

The main signal transduction pathway of GPR139 receptors remains unknown, but previous studies have reported signaling through G_i_ (Süsens et al., 2006), G_s_ (Hu et al., 2009), and particularly G_q_ (Matsuo et al., 2005; Shi et al., 2011; Isberg et al., 2014; Wang et al., 2015; Bayer Andersen et al., 2016) protein pathways. The endogenous aromatic L-amino acids L-tryptophan (a precursor of serotonin) and L-phenylalanine (a precursor of tyrosine) activate GPR139 receptors (Isberg et al., 2014; Wang et al., 2015). Interestingly, a recent study reported that tryptophan depletion was associated with compulsive-like behavior in rats (Merchán et al., 2017), thus indicating a possible link between GPR139 receptors and the compulsive-like behavior that was observed in the present study. Moreover, low plasma concentrations of tryptophan have been suggested to be correlated with alcohol abuse in humans (Virkkunen and Linnoila, 1990). Withdrawal from alcohol has also been shown to significantly decrease brain tryptophan concentrations (Bano et al., 1996). GPCR-interacting proteins are known to regulate the activity, trafficking, and localization of GPCRs (Magalhaes et al., 2012). Low plasma and brain tryptophan concentrations that are induced by chronic alcohol intake (Virkkunen and Linnoila, 1990; Bano et al., 1996) may thus alter the function and/or expression of GPR139 (e.g., through upregulation). Additionally, withdrawal from alcohol increases the expression of the transcription factor Fos in the habenula (Li et al., 2016), which also interacts with the GH16I020080 regulatory element of the *GPR139* gene, acting as an enhancer of the gene (Genecards, 2018). These alternative mechanisms may explain why the GPR139 receptor agonist was effective only in alcohol-dependent rats, specifically in the subgroup that had a compulsive-like phenotype and exhibited high alcohol intake. However, further studies are necessary to better investigate this phenomenon.

Experiments that characterize the pharmacology and function of GPR139 receptors and identify antagonist compounds with favorable pharmacokinetics *in vivo* are currently ongoing (Wang et al., 2015; Hitchock et al., 2016; Shehata et al., 2016; Nøhr et al., 2017). GPR139 receptor mRNA is abundantly expressed in the habenula, ventrolateral region of the caudate putamen, zona incerta, and medial mammillary nucleus. High and specific expression of the GPR139 receptor has been found in the habenula and septum in mice, with the highest immunoreactivity in the medial habenula (Matsuo et al., 2005; Wang et al., 2015; Hitchock et al., 2016). A low level of expression has also been detected in the IPN (Liu et al., 2015). The habenula is a central structure that regulates monoaminergic systems, notably dopamine and serotonin, and integrates cognitive, emotional, and sensory processing (Boulos et al., 2017). The habenula receives inputs from the basal ganglia, septum, hypothalamus, anterior cingulate cortex, and medial prefrontal cortex and projects to several midbrain regions, most importantly the IPN and rostromedial tegmental nucleus, regulating the activity of monoaminergic nuclei (Herkenham and Nauta, 1979; Bianco and Wilson, 2009). Thus, the habenula is a crucial intersection between cortical and subcortical structures that are implicated in emotion, stress, and reward processing (Batalla et al., 2017). Both the lateral and medial habenula have been implicated as parts of intrinsic reinforcement circuitry, making it an interesting target for addiction studies (Matsumoto and Hikosaka, 2007; Hsu et al., 2014; Velasquez et al., 2014). Emerging evidence suggests that medial habenula-IPN circuitry is critical in addiction and anxiety (McLaughlin et al., 2017). GPR139 receptors are predominantly expressed in the medial habenula. Therefore, we hypothesize that the reduction of alcohol self-administration in dependent rats that was observed in the present study may be mediated by habenular circuits. Importantly, we found that the local activation of GPR139 receptors in the habenula but not IPN reversed the escalation of alcohol self-administration in alcohol-dependent rats and decreased withdrawal-induced hyperalgesia, further indicating that the habenula is a mediator of the effects of GPR139 agonism on alcohol dependence.

In summary, the present study provided robust preclinical evidence that GPR139 receptor activation reverses compulsive-like alcohol drinking and decreases withdrawal-induced hyperalgesia in a subgroup of alcohol-dependent rats that exhibit symptoms of alcohol dependence. The reductions of alcohol self-administration and withdrawal-induced hyperalgesia were mediated by the habenula and not IPN. JNJ-63533054 is orally bioavailable and has a favorable pharmacokinetic profile. It selectively decreased alcohol intake in a subgroup of dependent rats that exhibited a compulsive-like phenotype, suggesting that this GPR139 receptor agonist may be a candidate for further drug development for the treatment of alcohol use disorder. Further studies are needed to determine the underlying mechanisms by which GPR139 receptors regulate compulsive-like alcohol drinking and mechanical hyperalgesia and whether targeting GPR139 receptors may also affect addiction-like behaviors with other drugs of abuse and/or non-drug-related compulsivity.

## Synthesis

Reviewing Editor: Karen Szumlinski, University of California at Santa Barbara Decisions are customarily a result of the Reviewing Editor and the peer reviewers coming together and discussing their recommendations until a consensus is reached. When revisions are invited, a fact-based synthesis statement explaining their decision and outlining what is needed to prepare a revision will be listed below. The following reviewer(s) agreed to reveal their identity: Rodrigo Leão.

Upon consultation with two expert reviewers, we commend the authors on a very solid and interesting paper and have reached the decision of “Revise and Re-review” as some very addressable concerns were raised with the original manuscript. Please see these concerns below. I look forward to the revised version of your report.

Reviewer 1:

Little is known about the GPR139 receptor, and especially little is known about its behavioral influences. Hence, identification of a dual role in pain and alcohol seeking is novel and interesting.

The authors have conducted a set of experiments examining the role of the orphan receptor GPR139 in alcohol addiction and withdrawal. They showed that a selective agonist at this receptor (JNJ 63533054) reverses escalation of alcohol drinking in dependent rats, blocks withdrawal-induced hyperalgesia (but not somatic signs), and was specifically effective only in a subset of high-compulsive rats.

Overall, the experiments are well designed, the results interesting, and the interpretations thoughtfully discussed with regard to possible roles of aversion and the medial habenula. I appreciated the discussion of locomotor confounds (or the apparent lack thereof) as well as the frank discussion of shortcomings in overall understanding of the GPR139 receptor, including the lack of a specific antagonist and the unclear signaling pathways of this receptor.

I saw only a few minor shortcomings, that are hopefully readily addressable:

1. The paw withdrawal scores are measured in withdrawn animals with and without JNJ, but not in alcohol-naïve animals. Without such a group, it is difficult to interpret the magnitude of the effect of JNJ - did it largely restore pain thresholds back to ‘normal’? Or was the effect only partial?

2. It was curious that the paw withdrawal scores were not analyzed separately for the high-compulsive and low-compulsive animals. Was there not enough statistical power to do this analysis? It would certainly strengthen the authors' case if the effects of JNJ on pain scores were, like the effects on alcohol drinking, stronger in the high-compulsive animals.

3. Category labels appear to be missing from the bar graphs in Fig. 5A. Presumably, they are the same as in Fig. 2A.

Reviewer 2:

The authors had used a chronic intermittent exposure (CIE) to alcohol vapor combined with operant self-administration that it is a sophisticated protocol to develop dependence in rodents and is very useful to compare behavioral differences in between non-dependent and dependent rat. The GPR139 is an orphan G protein-coupled receptor that is expressed primarily in the brain. Not much is known regarding the function of GPR139. The authors showed results providing robust preclinical evidence that GPR139 receptor activation reverses key addiction-like behaviors in dependent animals. In this sense, GPR139 might be a novel target for the treatment of alcohol use disorder. I think the manuscript makes an important and useful contribution to the literature.

In this manuscript, the authors report on studies examining if the modulation of GPR139 may be relevant to alcohol addiction-related behaviors. In the study they tested the effects of systemic administration and microinjection (in the habenula and interpeduncular nucleus) of a selective GPR139 receptor agonist (JNJ 63533054), on alcohol self-administration seeking behavior and alcohol withdrawal-induced hyperalgesia in alcohol-dependent rats. Surprisingly, the authors found that activation of GPR139 in the habenula, but not interpeduncular nucleus, reduced both alcohol self-administration and withdrawal-induced hyperalgesia in dependent rats. In my opinion the work is novel and has important implications suggesting that GPR139 may be a novel target for the treatment of alcohol use disorder. The experimental designs and statistical analyses were appropriate. They discuss caveats with their repeated behavioral testing. Overall: This was all very well done. The manuscript is well written, the data are well presented and discussed. And this paper addresses an interesting and important topic. However, there are few major concerns that need to be addressed.

#1 - In the discussion it is not very clear an explanation to observe the decrease of the alcohol seeking behavior and alcohol withdrawal-induced hyperalgesia just for dependent and not for non-dependent rats.

#2 - While the GPR139 receptor mRNA is abundantly expressed in the habenula

and in other different brain regions, the significance of a possible alteration in the level of GPR139 receptors after the development of alcohol dependence should be discussed.
